# Acute Diffuse Renal Tubulopathy in a Patient With Lung Cancer: A Case Report

**DOI:** 10.3389/fmed.2021.742489

**Published:** 2021-10-04

**Authors:** Po-Jung Tseng, Ming-Tso Yan

**Affiliations:** ^1^Division of Nephrology, Department of Medicine, Cathay General Hospital, School of Medicine, Fu-Jen Catholic University, Taipei, Taiwan; ^2^National Defense Medical Center, Graduate Institute of Medical Sciences, Taipei, Taiwan

**Keywords:** drug therapy, acute kidney injury, comorbidity, creatinine, electrolytes, pembrolizumab, immune checkpoint inhibitors, case report

## Abstract

Immune checkpoints inhibitors (ICPIs), as either a frontline or adjuvant therapy, showed favorable outcomes among diverse malignancies. Immune-related adverse events (IRAEs) are increasingly encountered, but the kidneys are rarely affected. A 67-year-old man with stage IV squamous cell carcinoma of the lung presented with acute kidney injury and hypercalcemia secondary to bone metastasis. After an aggressive saline infusion and subcutaneous denosumab 60mg administration, his renal function and serum calcium level were recovered on day 4. Due to his intolerance to chemotherapy, immunotherapy with a monoclonal antibody targeting programmed cell death protein-1 (PD-1), pembrolizumab 2mg/kg, was used on day 4. On day 11, polyuria, non-albumin dominant proteinuria, and severe deficiencies of electrolytes (potassium 2.5 mmol/L, calcium 5.5 mg/dL, magnesium 1.3 mg/dL, and phosphate 1.5 mg/dL) along with concomitant renal wasting were developed acutely. Except for postponing the next pembrolizumab, prednisolone at 1 mg/kg/day was given on day 13. On day 27, his polyuria subsided and urine protein loss resolved. Serum levels of potassium, phosphate, calcium, and magnesium all returned within the reference range. This case highlighted that renal IRAEs, even though uncommon, could be severe and potentially life-threatening if left unrecognized and untreated. Early recognition of renal IRAEs and prompt withdrawal of ICPIs may result in lower renal morbidity.

## Introduction

Immune checkpoints are composed of different pathways functioning to maintain self-tolerance and modify immune response. Recent progress in understanding that tumor cells could hijack these pathways to evade immune elimination urged the development of immune therapies targeting immune checkpoints (1). Immune checkpoints inhibitors (ICPIs) include monoclonal antibodies blocking either programmed cell death protein-1 (PD-1)/programmed death-ligand 1 (PD-L1) pathway or cytotoxic T-lymphocyte associated protein 4 (CTLA-4)/CD28 axis (2). Favorable outcomes have been increasingly reported in using ICPIs among diverse malignancies and approval indications have been expanding rapidly in either frontline or adjuvant settings. With the increasing use of ICPIs, the lurking threats of ICPIs related toxicity became widely recognized, especially immune-related adverse events (IRAEs) (3).

The spectrum of clinical manifestations in IRAEs is broad because a number of organs could be affected by immune intolerance including skin, gastrointestinal tract, lung, heart, endocrine organs, and nervous system being (4). Pembrolizumab is one of PD-1 inhibitors and, similar to other ICPIs, its IRAEs rarely affect the kidneys (5). However, pembrolizumab-associated proteinuria, acute kidney injury, and rarely renal tubular acidosis are increasingly reported recently (6, 7). To the best of our knowledge, pembrolizumab or other PD-1 inhibitors have not been published to cause a diffuse renal tubulopathy-related lethally acid-base and electrolytes disturbance. In this article, we present a case of developing a life-threatening electrolytes disturbance after receiving pembrolizumab therapy.

## Case Description

A 67-year-old man presented with 1 week of fatigue, abdominal bloating, and constipation. He was found to have stage IV squamous cell carcinoma of the lung with bone metastasis 4 months ago, for which he received the first cycle of gemcitabine combined with cisplatin until 4 weeks ago. He denied a prior history of systemic diseases.

On admission, his blood pressure was 123/82 mmHg, pulse rate 102 beats/min, respiratory rate 18/min, and body temperature 37°C. Physical examination revealed poor skin turgor and mildly distended abdomen with tympanic percussion. The remainder of the physical examination was unremarkable. Laboratory tests showed an elevated serum creatinine (2.8 mg/dL; baseline 0.7 mg/dL 4 weeks ago), hypercalcemia (corrected Ca^2+^ 12.6 mg/dL), hyperphosphatemia (5.8 mg/dL) and a normal parathyroid hormone (PTH) level (23.7 pg/mL) (Table 1). Intravenous saline and subcutaneous denosumab 60 mg were used for treating hypercalcemia. On day four, his serum creatinine and Ca^2+^ was recovered. Due to chemotherapy intolerance, therapy was switched to immunotherapy with programmed cell death protein-1 (PD-1) receptor blocker, pembrolizumab 2mg/kg. On day 11, his urine output acutely increased to 4 to 5 L/day. He developed severe electrolytes disturbance, including hypokalemia (2.5 mmol/L), hypocalcemia (5.5 mg/dL), hypomagnesemia (1.3 mg/dL) and hypophosphatemia (1.5 mg/dL) along with renal wasting of these electrolytes (urine K^+^/Cr 14.5 mmol/mmol, urine Ca^2+^/Cr 0.39 mg/mg, fractional excretion (FE) of Mg^2+^ 53% and FE of phosphate 110%, respectively). He also had a new-onset of non-albumin predominant proteinuria (1,064 mg in 24-h urine collection) and persistent isothenuria in the absence of a deteriorated renal function (Table 1). Aggressive replacement of deficient electrolytes orally and intravenously was performed for 2 days but treatment was not successful. On day 13, oral prednisolone 1 mg/kg/day was immediately initiated along with discontinuation of pembrolizumab. On day 27, the serum levels of K^+^, phosphate, Ca^2+^, and Mg^2+^ all returned within the reference range and daily urine output decreased from 4–3 L to 1.0–1.5 L/day (Table 2).

**Table 1:** Changes of laboratory values.

**Items**	**Unit**	**Reference**	**4 weeks ago**	**Admission**	**Day 4**	**Day 11**
Serum						
BUN	mg/dl	8–25		66	18	12
Cr	mg/dl	0.63–1.30	0.7	2.80	0.75	0.59
Na^+^	mmol/l	135–145		127	134	141
K^+^	mmol/l	3.5–5.3		4.9	3.9	2.5
Ca^2+^	mg/dl	8.5–10.0		12.6	9.8	5.5
Phosphate	mg/dl	2.5–4.5		5.8	3.4	1.5
Mg^2+^	mg/dl	1.6–2.3		2.7		1.3
iPTH	pg/ml	8.0–76.0		23.8		
Urine						
U${}_{\text{K}}^{+}$/Cr	mmol/mmol					14.5
UCa^2+^/Cr	mg/mg					0.39
FE${}_{\text{Na}}^{+}$	%					1.1
FE${}_{\text{Mg}}^{2+}$	%					53.0
FE_phosphate_	%					110.0
Protein	mg/day					1,064.0
Osmolality						273
Amount	ml					4,800

*BUN, blood urea nitrogen; FE, fractional excretion; iPTH, intact parathyroid hormone*.

**Table 2:** Laboratory data after 16-day withdrawal of pembrolizumab.

**Items**	**Unit**	**Reference**	**Day 27**
Serum			
BUN	mg/dl	8–25	15
Cr	mg/dl	0.63–1.30	0.42
Na^+^	mmol/l	135–145	138
K^+^	mmol/l	3.5–5.3	3.6
Ca^2+^	mg/dl	8.5–10.0	8.6
Phosphate	mg/dl	2.5–4.5	2.5
Mg^2+^	mg/dl	1.6–2.3	1.5

## Discussion

The presence of polyuria, isosthenuria, a new onset non-albumin predominant proteinuria, and severe disturbance of serum electrolytes including hypokalemia, hypocalcemia, hypophosphatemia, and hypomagnesemia with inappropriate renal wasting of K^+^, Ca^2+^, Mg^2+^, and phosphate suggests diffuse renal tubular injury. In the absence of other acquired tubulopathy causes such as systemic diseases, diuretics or the use of nephrotoxic agents, pembrolizumab-induced diffusely renal tubulopathy was favored.

ICPIs have revolutionized cancer treatments, either as first-line therapies or as adjuvant therapies. Tumors are able to evade immunosurveillance *via* diverse mechanisms including induction of immune tolerance through activating immune checkpoints (8). Pembrolizumab is one of the ICPIs targeting PD-1 on T lymphocytes to hinder the binding between PD-1 and its ligands (9). The prevalence of pembrolizumab-associated IRAEs, like other ICPIs, was reported higher than 50% but kidneys were rarely affected (7). However, with the increasing experience of pembrolizumab use, it has been proposed that the prevalence of pembrolizumab-associated renal toxicities may be underestimated (10). The most common renal toxicity of pembrolizumab is acute kidney injury (AKI) and the incidence has been reported ~1.7–6.7% with acute tubular injury or acute interstitial nephritis (AIN) being the most common pathological findings (5, 6, 11). In contrast, glomerular diseases are less frequent renal IRAEs, mainly podocytopathy. Distal renal tubule acidosis, although rare, was also identified in some case reports (12). However, diffusely renal tubule damages with severe electrolytes deficiency in the absence of AKI, as presented in our case study, have not been described before. In addition to assess renal function, our case highlights that the levels of serum electrolytes may warrant a close monitor after pembrolizumab therapy to prevent unrecognizing life-threatening electrolytes disturbance.

Denosumab, a monoclonal antibody directly against receptor activator of nuclear factor-κB ligand (RANKL), is widely used in osteoporosis or bone metastasis. Denosumab can cause hypocalcemia with a mechanism similar to hungry bone syndrome (13) as well as hypophosphatemia due to renal phosphate losing which is mediated by hypocalcemia-related secondary hyperparathyroidism post denosumab injection and is also a hungry bone-like mechanism (14). The hypophosphatemia with renal phosphate wasting in our case is indistinguishable from those caused by denosumab. However, denosumab has not been reported to cause hypokalemia, hypomagnesemia, proteinuria, or impaired urine concentration ability (15). Besides, urine calcium excretion will be decreased during denosumab-related hypocalcemia. Accordingly, both onset and recovery of each electrolyte's disturbances with renal wasting and impaired concentration ability occurred concomitantly, which suggested that pembrolizumab may be the main culprit.

Some risk factors of renal IRAEs have been identified including the use of proton pump inhibitors, impaired renal function, and combined use of ICPIs (16). The mechanism of pembrolizumab-associated kidney injury remains unclear. The ligand of PD-1 (PD-L1) expressed on renal epithelial cells has been reported to protect the kidneys against ischemia-reperfusion injury via inducing immune tolerance, regulating the activation of different T cell subgroups, and modifying cytokines and chemokines (11, 17). The use of pembrolizumab soon after AKI in this patient may interrupt PD-1/PD-L1 interaction-related immune tolerance, and cause autoimmune response and dysregulation of post-AKI inflammation; therefore, leading to tubulointerstitial damages (17). However, the wide variation in clinicopathological presentations of ICPI-related nephropathy suggests a more complex mechanism. Recently, the RANK-RANKL pathway was discovered to be crucial in many immune processes such as regulatory T lymphocytes generation (17). The synergistic anti-tumor effects of denosumab were described during the combination with ICPIs (18, 19). Therefore, denosumab needs to be accessed due to the possible increased risks of ICPIs related IRAEs since the synergistic effects may aggravate the autoimmune response and dysregulation of inflammation in the injured kidneys of our patient.

Discontinuation of PD-1 inhibitors is imperative when severe adverse kidney effects occur. Temporary use of immunosuppressant agents is common regarding the potentially pathogenic role of activation of autoreactive lymphocytes by PD-1 inhibitors (20). Except for glucocorticoid, several immunosuppressant agents were investigated including mycophenolate mofetil, cyclophosphamide, and rituximab. Infliximab against tumor necrosis factor-α (TNF-α) was found to be effective while patients had a poor response to other immunosuppressant agents, which is possibly because TNF-α is upregulated during ICPIs treatment (21). In general, a favorable renal prognosis has been reported in pembrolizumab-induced nephrotoxicities if diagnosis and management are prompt, best illustrated by the complete recovery of renal function in our patient. The rapid recovery of renal IRAEs within 14 days in our patient may suggest the role of immunosuppressant agents, supported by the reports with recovery to baseline kidney function within 1 month under the immunosuppressant therapy (22).

In conclusion, renal IRAEs, albeit uncommon, may be severe and potentially life-threatening if left unrecognized and untreated. Our case highlighted that early recognition along with the prompt withdrawal of culprit ICPIs helps to achieve a better outcome.

## Data Availability Statement

The original contributions presented in the study are included in the article/supplementary material, further inquiries can be directed to the corresponding author.

## Author Contributions

P-JT collected data and made the tables. P-JT and M-TY drafted and revised the paper. Both authors approved the final manuscript.

## Conflict of Interest

The authors declare that the research was conducted in the absence of any commercial or financial relationships that could be construed as a potential conflict of interest.

## Publisher's Note

All claims expressed in this article are solely those of the authors and do not necessarily represent those of their affiliated organizations, or those of the publisher, the editors and the reviewers. Any product that may be evaluated in this article, or claim that may be made by its manufacturer, is not guaranteed or endorsed by the publisher.

## References

[1] Li B Chan HL Chen P. Immune checkpoint inhibitors: basics and challenges. Curr Med Chem. (2019) 26:3009–25. 10.2174/0929867324666170804143706 28782469

[2] Darvin P Toor SM Sasidharan Nair V Elkord E. Immune checkpoint inhibitors: recent progress and potential biomarkers. Exp Mol Med. (2018) 50:1–11. 10.1038/s12276-018-0191-1 30546008 PMC6292890

[3] Postow MA Sidlow R Hellmann MD. Immune-related adverse events associated with immune checkpoint blockade. N Engl J Med. (2018) 378:158–68. 10.1056/NEJMra1703481 29320654

[4] Ramos-Casals M Brahmer JR Callahan MK Flores-Chávez A Keegan N Khamashta MA. Immune-related adverse events of checkpoint inhibitors. Nat Rev Dis Primers. (2020) 6:38. 10.1038/s41572-020-0160-6 32382051 PMC9728094

[5] Herrmann SM Perazella MA. Immune Checkpoint Inhibitors and Immune-Related Adverse Renal Events. Kidney Int Rep. (2020) 5:1139–48. 10.1016/j.ekir.2020.04.018 32775813 PMC7403510

[6] Izzedine H Mathian A Champiat S Picard C Mateus C Routier E. Renal toxicities associated with pembrolizumab. Clin Kidney J. (2019) 12:81–8. 10.1093/ckj/sfy100 30746132 PMC6366307

[7] Cortazar FB Marrone KA Troxell ML Ralto KM Hoenig MP Brahmer JR. Clinicopathological features of acute kidney injury associated with immune checkpoint inhibitors. Kidney Int. (2016) 90:638–47. 10.1016/j.kint.2016.04.008 27282937 PMC4983464

[8] Dyck L Mills KHG. Immune checkpoints and their inhibition in cancer and infectious diseases. Eur J Immunol. (2017) 47:765–79. 10.1002/eji.201646875 28393361

[9] Baxi S Yang A Gennarelli RL Khan N Wang Z Boyce L. Immune-related adverse events for anti-PD-1 and anti-PD-L1 drugs: systematic review and meta-analysis. BMJ. (2018) 360:k793. 10.1136/bmj.k793 29540345 PMC5851471

[10] Wanchoo R Karam S Uppal NN Barta VS Deray G Devoe C. Adverse renal effects of immune checkpoint inhibitors: a narrative review. Am J Nephrol. (2017) 45:160–9. 10.1159/000455014 28076863

[11] Perazella MA. Checkmate: kidney injury associated with targeted cancer immunotherapy. Kidney Int. (2016) 90:474–6. 10.1016/j.kint.2016.05.024 27521108

[12] El Bitar S Weerasinghe C El-Charabaty E Odaimi M. Renal tubular acidosis an adverse effect of PD-1 inhibitor immunotherapy. Case Rep Oncol Med. (2018) 2018:8408015. 10.1155/2018/8408015 29666732 PMC5831873

[13] Dave V Chiang CY Booth J Mount PF. Hypocalcemia post denosumab in patients with chronic kidney disease stage 4-5. Am J Nephrol. (2015) 41:129–37. 10.1159/000380960 25790847

[14] Megapanou E Florentin M Milionis H Elisaf M Liamis G. Drug-induced hypophosphatemia: current insights. Drug Saf. (2020) 43:197–210. 10.1007/s40264-019-00888-1 31776845

[15] Suzuki T Nakamura Y Kato H. Changes of bone-related minerals during DENOSUMAB administration in post-menopausal osteoporotic patients. Nutrients. (2017) 9:871. 10.3390/nu9080871 28805705 PMC5579664

[16] Cortazar FB Kibbelaar ZA Glezerman IG Abudayyeh A Mamlouk O Motwani SS. Clinical Features and outcomes of immune checkpoint inhibitor-associated AKI: a multicenter study. J Am Soc Nephrol. (2020) 31:435–46. 10.1681/ASN.2019070676 31896554 PMC7003302

[17] Perazella MA Shirali AC. Nephrotoxicity of cancer immunotherapies: past, present and future. J Am Soc Nephrol. (2018) 29:2039–52. 10.1681/ASN.2018050488 29959196 PMC6065079

[18] Liede A Hernandez RK Wade SW Bo R Nussbaum NC Ahern E. An observational study of concomitant immunotherapies and denosumab in patients with advanced melanoma or lung cancer. Oncoimmunology. (2018) 7:e1480301. 10.1080/2162402X.2018.1480301 30524886 PMC6279336

[19] Tan W Zhang W Strasner A Grivennikov S Cheng JQ Hoffman RM. Tumour-infiltrating regulatory T cells stimulate mammary cancer metastasis through RANKL-RANK signaling. Nature. (2011) 470:548–53. 10.1038/nature09707 21326202 PMC3166217

[20] Michot JM Bigenwald C Champiat S Collins M Carbonnel F Postel-Vinay S. Immune-related adverse events with immune checkpoint blockade: a comprehensive review. Eur J Cancer. (2016) 54:139–48. 10.1016/j.ejca.2015.11.016 26765102

[21] Murakami N Borges TJ Yamashita M Riella LV. Severe acute interstitial nephritis after combination immune-checkpoint inhibitor therapy for metastatic melanoma. Clin Kidney J. (2016) 9:411–7. 10.1093/ckj/sfw024 27274826 PMC4886917

[22] Shirali AC Perazella MA Gettinger S. Association of acute interstitial nephritis with programmed cell death 1 inhibitor therapy in lung cancer patients. Am J Kidney Dis. (2016) 68:287–91. 10.1053/j.ajkd.2016.02.057 27113507

